# Dynamic Consolidation Measurements in a Well Field Using Fiber Bragg Grating Sensors

**DOI:** 10.3390/s19204403

**Published:** 2019-10-11

**Authors:** Sandra Drusová, R. Martijn Wagterveld, Adam D. Wexler, Herman L. Offerhaus

**Affiliations:** 1 Wetsus, European Centre of Excellence for Sustainable Water Technology, Oostergoweg 9, 8911 MA Leeuwarden, The Netherlands; martijn.wagterveld@wetsus.nl (R.M.W.); adwexler@u.washington.edu (A.D.W.); 2 Optical Sciences, University of Twente, Hallenweg 23, 7522 NH Enschede, The Netherlands; h.l.offerhaus@utwente.nl

**Keywords:** fiber Bragg grating, consolidation, groundwater extraction, FBG packaging

## Abstract

Currently available groundwater flow prediction tools and methods are limited by insufficient spatial resolution of subsurface data and the unknown local heterogeneity. In this field study, fiber Bragg grating (FBG) sensors were installed in an extraction well field to investigate its potential to measure groundwater flow velocity. Reference in-situ pore pressure and temperature measurements were used to identify possible sources of FBG responses. FBG strain sensors were able to detect soil consolidation caused by groundwater extraction from 250 m distance. The results show that FBG responses were influenced by interface friction between soil and FBG packaging. FBG packaging slipped in soil and the effect was more pronounced during higher groundwater flow around a nearby well. These FBG fibers could be applied for indirect flow monitoring that does not require any tracer and provide real-time and long-term data during regular operation of extraction wells.

## 1. Introduction

Groundwater is stored in porous sediments in the subsurface (aquifers). Large volumes of water are moving in aquifers with velocities in the order of a few μm s^−1^ to hundreds of μm s^−1^. Due to large residence time in aquifers, groundwater is filtered from pollutants and presents a naturally cleaned source of drinking water or irrigation water. Groundwater supply is brought to the surface through wells. Understanding and long-term monitoring of aquifers and wells, especially groundwater flow, is crucial for sustainable management of groundwater resources and prevention of groundwater-related problems, e.g., overpumping, clogging, and groundwater pollution. Having accurate long-term flow information near wells helps to identify wells where clogging will most likely occur and to choose suitable locations for new wells.

Groundwater flow velocity and direction can be either obtained directly by various types of flowmeters installed inside the observation wells, or indirectly from geological modeling tools or geoelectrical imaging [1,2]. Common input to the models are field data from tracer tests, borehole logs, hydraulic head measurements and pumping tests. Interpreting data from groundwater flowmeters is not straightforward because the well itself causes a disturbance to the flow. The construction of a well may create preferential flows around it and the shape of the well screen influences vertical flow in the well itself [3]. Groundwater flow models are a useful tool to understand the overall flow distribution, but they are still based on simplified assumptions and data from a limited number of monitoring wells. For heterogeneous subsurface environments, groundwater models cannot accurately predict local flow distribution. The models can be improved by more accurate input data with higher spatial resolution.

The most recent technology that can provide higher resolution groundwater flow data is optical fiber sensors. Fiber sensors are small in size, can be installed in direct contact with the soil to minimize the disturbance to groundwater flow. Current state-of-the art technology for monitoring groundwater flow is Distributed Temperature Sensing (DTS) [4]. DTS is based on inelastic scattering in an optical fiber which is sensitive to temperature. The entire length of the fiber is a sensing element providing data with medium spatial and high temperature resolution (currently 12.5 cm and 0.01 °C). Heat is introduced into the subsurface as a tracer for the flow [5] either via hot water injection [6,7] or directly heating up the subsurface [8,9]. The accuracy of heat tracing methods needs to be discussed because warming up the subsurface changes properties of water and potentially modifies the flow patterns [10]. Recent setups combine the heating element directly with the DTS fiber-optic cable referred to as Active Heating-DTS [11,12].

In our previous study [13], we described the possibilities for groundwater flow sensing using another type of optical fiber sensors, Fiber Bragg Grating (FBG) sensors. An FBG sensor is a periodic modulation of refractive index in a fiber core. This grating reflects light with wavelength $\lambda_{0}$ (Bragg wavelength) matching the period of the grating Λ (Bragg condition):

(1) $$\lambda_{0}=2n_{eff}\Lambda$$

where $n_{eff}$ is effective refractive index for the propagating light. Gratings are typically shorter than 1 mm, meaning that FBG sensors are localized measurement points in an optical fiber. A shift in the Bragg wavelength Δλ is a result of a modification of refractive index or grating period, either by temperature change ΔT or strain Δ*ϵ* in the optical fiber:

(2) $$\frac{\Delta \lambda }{\lambda_{0}}=\left(\alpha_{e}+\alpha_{n}\right)\Delta T+\left(1-p_{e}\right)\Delta \epsilon$$

where $p_{e}$ is a strain–optic coefficient, $\alpha_{e}$ is a thermal expansion coefficient, and $\alpha_{n}$ is a thermo-optic coefficient. FBG sensors are sensitive to multiple physical processes at the same time (temperature, pressure, and deformation forces). FBG sensors installed in the direct contact with soil can measure soil deformation caused by groundwater extraction from an aquifer [14,15,16]. Various types of FBG extensometers have been tested to monitor natural land subsidence [17] or subsidence caused by mining [18]. In these studies, however, drilled boreholes with extensometers were not refilled with the original soil.

Volumetric changes in soil caused by vertical loading are known under term consolidation [19,20]. Groundwater extraction decreases pore pressure in saturated soil, which can be measured by piezometers in monitoring wells. Lowering pore pressure results in an increase in vertical stress in an aquifer and subsequent reduction of pore volume and compaction of the soil. Consolidation is therefore coupled to groundwater flow via pore pressure changes. The first coupled theory was developed by Biot [21]. In current hydrological applications, groundwater flow is modeled separately and pore pressure distribution is then used as the external driving force for the consolidation [14,19]. High resolution consolidation data collected from an examined site could also provide an input for the flow models.

Our previous research [13] showed that FBG sensors with a standard polyvinyl chloride (PVC) and teflon packaging already respond to pressure and flow changes in an aquifer simulator with natural flow. Pressure changes could be related to consolidation, while the mechanism of possible flow coupling is less clear. In this field study, FBG sensors with teflon packaging were deployed in a drinking water well field to investigate new methods of measuring groundwater flow. The packaging was intentionally not adapted to measure only consolidation strain. Response of FBG sensors is discussed for two different groundwater flow cases with multiple wells involved in the extraction.

## 2. Materials and Methods

**Figure 1:**
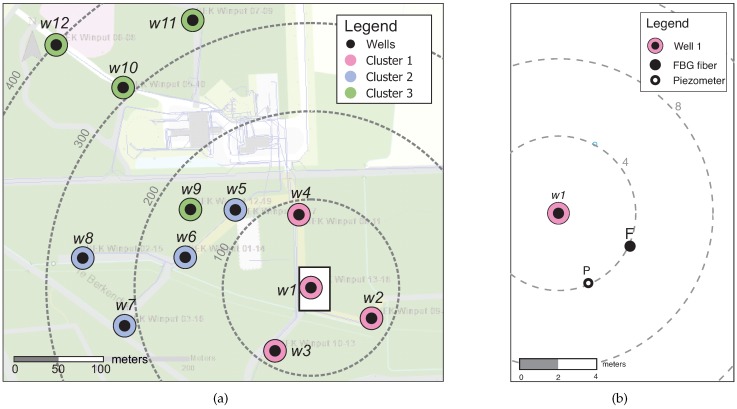
A map of the drinking water well field: (**a**) Location of the extraction wells. The area in the white rectangle is displayed in (**b**). (**b**) Location of FBG fiber and reference piezometer near well 1.

The study was conducted in a drinking water extraction field in Hengelo, Gelderland, The Netherlands. Here, groundwater is extracted from an unconfined aquifer from depths of 15–34 m. Soil borehole logs show mostly sand, gravel and, occasionally, loam layers. The well field contains 12 extraction wells with a maximum flowrate of 130 m^3^ h^−1^ for each well. The extraction wells are divided into three clusters with four wells in each cluster (see Figure 1a). During regular operation three pumps extract simultaneously.

**Figure 2:**
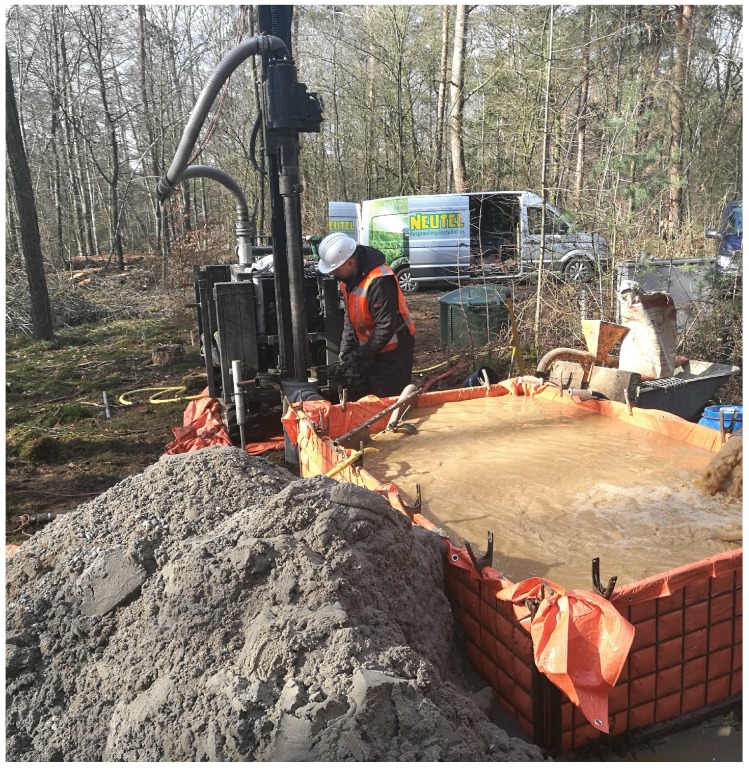
Drilling of boreholes for FBG fibers and piezometer.

Two types of sensors were installed in close proximity of well w1 (Figure 1b): Bundles of fiber Bragg grating (FBG) sensors, and reference divers (pressure and temperature sensors). Boreholes of 30 cm diameter were suction drilled 38 m deep (see Figure 2). w1 has a well screen at a depth of 17.8–32 m.

The fiber bundle was glued using silicon to a nylon weighted fishing line to keep it stretched while being lowered in the borehole. The borehole was refilled with the mixture of the extracted sediment, the top part was filled with gravel. In this way, the placement of fibers caused minimal disturbance to the aquifer.

PVC tubes with 2 m long thin slits (screens) were installed in the boreholes as piezometer tubes. The borehole was refilled with the mixture of the original material and the slit area was sealed with clay. A reference diver (TD reference diver, van Essen Instruments, Delft, the Netherlands) was installed in the piezometer tube, 10 m below the surface.

Fiber bundle *F* consisted of three optical fibers placed in a teflon tube for protection (3 mm diameter, 1 mm thick). Each optical fiber contains eight FBG sensors with wavelengths 1518, 1527, 1536, 1545, 1554, 1563, 1572 and 1581 nm, a total of 24 sensor per bundle. FBG sensors were written using UV-laser in a standard single mode SMF28 fiber with an acrylate coating (Loptek, Berlin, Germany). The FBG sensors were 0.7 m apart and covered the range of 17–33 m, corresponding to the screen depth of the nearest well, *w*1.

An autonomous sensing unit was assembled to collect and transfer data from the field location. A microcontroller (Raspberry Pi model 3B, Raspberry Pi Foundation, UK) handled the communication with the FBG interrogator (Hyperion si155, LUNA, USA), optical switch (eol 1 × 16, National Instruments), weather station (Dreamlink WH1080, Conrad, Germany), and data server. The data were transferred via 3G mobile connection.

The FBG interrogator has a built-in wavelength reference to guarantee long-term accuracy of the measurements. The interrogator’s internal peak detection algorithm identifies the peak Bragg wavelengths from the spectrum 1500–1600 nm with a scanrate of 100 Hz. The interrogator was synchronized with an optical switch. An idle fiber was addressed before addressing each sensor fiber to improve detection. Each FBG sensor was sampled with a 2.1 s period.

The diver measures temperature and absolute pressure $p_{diver}$, which is a combination of pore pressure *p* and atmospheric pressure $p_{atm}$. Diver data were stored every 10 s in an internal memory and had to be collected manually. The weather station measured atmospheric pressure every 60 s. Pore pressure data for comparison with FBG responses were obtained from the reference diver and weather station:

(3) $$p=p_{diver}-p_{atm}$$

## 3. Results and Discussion

In this field study, FBG sensors were deployed in a drinking water well field to investigate the response upon well extraction. The FBG responses were measured during regular operation of the well field. The FBG sensors measure local temperature and strain variations in the installed fibers, which can be detected as a wavelength shift Δλ of the reflected light. The strain variation can be caused by several physical effects taking place in a set of coupled domains. FBG response in the well field can be written after adapting the general equation (Equation (2)):

(4) $$\frac{\Delta \lambda }{\lambda_{0}}=\alpha \Delta T+\left(1-p_{e}\right)\beta \Delta \epsilon_{c}+\left(1-p_{e}\right)\gamma \Delta \epsilon_{p}$$

The first term of FBG response is a temperature (ΔT) contribution, the second term is a consolidation strain ($\Delta \epsilon_{c}$) contribution and the third one is a pore pressure strain ($\Delta \epsilon_{p}$) contribution. α,β,γ are transfer coefficients which describe the influence of FBG packaging. $p_{e}$ is a strain–optic coefficient.

1. Temperature
  The temperature variation is expected to be minor in the investigated depth as groundwater temperature is very stable over the years. However, extraction mobilizes water from different depths and thus FBG sensors can respond to a temperature change ΔT. The temperature from the liquid is coupled through the teflon tube that is housing the fiber-bundle. The temperature sensitivity coefficient is in this case *α* = 7.43 × 10^−6^ K^−1^ [13], and temperature contribution to FBG response is
  (5) $$\frac{\Delta \lambda_{T}}{\lambda_{0}}=\alpha \Delta T$$
2. Consolidation
  The extraction of water can cause consolidation strain in the soil $\Delta \epsilon_{c}$ (−), which can be expressed as a function of pore pressure change Δp (Pa) [22].
  (6) $$\Delta \epsilon_{c}=\frac{\left(1+\nu_{soil}\right)\left(1-2\nu_{soil}\right)\Delta p}{\left(1-\nu_{soil}\right)E_{soil}}$$
  The values of Poisson’s ratio $\nu_{soil}$ and Young’s modulus $E_{soil}$ change with depth. For depth considered in our study, estimated values for dense fine sand are $E_{soil}$ = 50 MPa and $\nu_{soil}$ = 0.3 [23,24].
  FBG fibers compress/expand together with the surrounding soil and FBG sensors measure mechanical strain coupled to wavelength shift through strain–optic coefficient $p_{e}$ = 0.22. The strain experienced by FBG sensors is affected by the sensor packaging, therefore a consolidation strain transfer coefficient β (−) is introduced. Consolidation strain contribution to FBG response is
  (7) $$\frac{\Delta \lambda_{c}}{\lambda_{0}}=\left(1-p_{e}\right)\beta \Delta \epsilon_{c}$$
3. Pore pressure
  External pressure causes a volumetric change of the fiber which is coupled through the teflon tube and fiber coating.
  Increasing pressure compresses the material and decreases the grating period, thus wavelength shift is negative. FBG strain response to a pressure change Δp (Pa) is given in [25]. For glass, $\nu_{glass}=$0.17 is Poisson’s ratio and $E_{glass}=$ 73 GPa [26] is Young’s modulus:
  (8) $$\Delta \epsilon_{p}=-\frac{\Delta p(1-2\nu_{glass})}{E_{glass}}$$
  Pore pressure contribution to FBG response is then
  (9) $$\frac{\Delta \lambda_{p}}{\lambda_{0}}=\left(1-p_{e}\right)\gamma \Delta \epsilon_{p}$$
  where γ is a pressure transfer coefficient through the teflon tube and fiber coating.
4. Drag force
  The installed fiber-bundle might be in contact with flowing water instead of soil. The drag force of the flow can lead to bending of the fiber. According to Cadusch et al. [27], FBG sensor bent to 8 mm diameter measures 0.1 pm wavelength shift which is under the noise limit of the currently used FBG interrogator (1 pm). Drag force effects are a negligible source of FBG strain in our measurement setup.

The final response is a the result of all contributions and a coupling efficiency. Measured FBG responses from fiber F were compared to estimated FBG responses. Estimated FBG responses are calculated from pore pressure and temperature data collected from the reference piezometer P (for location, see Figure 1b). Temperature graphs are not shown because the contribution to the FBG response is not significant (see Table 1). Due to close proximity of the installed FBG fiber to well w1, extraction from this well has a major effect on the FBG sensor response. We can identify two situations that are discussed separately—with and without extraction from $w_{1}$.

### 3.1. Operation without Extraction from Well w1

**Figure 3:**
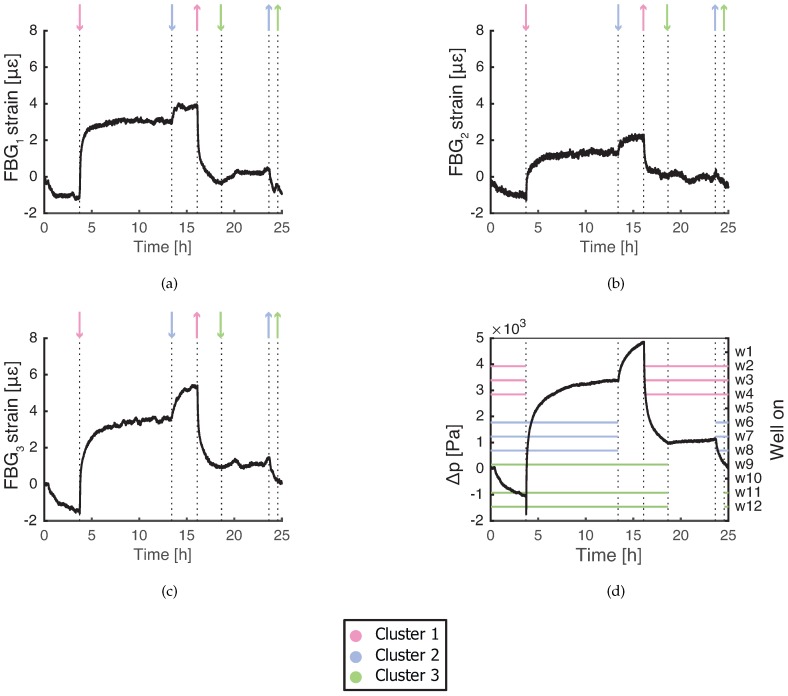
Measured strain response of FBG sensors during regular well operation with $w_{1}$ off: (**a**) $FBG_{1}$ at depth of 17.7 m; (**b**) $FBG_{2}$ at depth of 18.4 m; and (**c**) $FBG_{3}$ at depth of 19.1 m. (**d**) Pore pressure change in the reference piezometer in the 17.8–19.8 m depth range. ↑, a cluster of a corresponding color starts extracting; ↓, a cluster of a corresponding color stops extracting.

Without $w_{1}$ extracting, all FBG sensors in Figure 3a–c follow the pore pressure changes in Figure 3d. Pore pressure changes can be coupled to FBG strain through material deformation of the fiber and packaging or through soil consolidation. According to estimated values from Table 1, volumetric changes of the fiber due to pore pressure are a negligible contribution to FBG response, as well as temperature. FBG sensors in the current configuration measure consolidation strain in soil due to groundwater extraction.

The amplitude of the FBG response decreases with the distance to the wells which started/stopped the extraction (see Figure 3a–c). Wells from Cluster 1 cause the largest response, while wells from Cluster 2 cause smaller but detectable response. Influence of wells from the Cluster 3 is visible only for $FBG_{1}$ and $FBG_{3}$. FBG sensors detect consolidation strain caused by groundwater extraction up to 250 m distance (distance of the furthest well $w_{8}$).

Assuming that consolidation strain is constant within the depth span of the presented FBG sensors (1.4 m), expected values of consolidation strain transfer coefficient β are calculated in Table 2. The strain measured by the FBG sensors is one order of magnitude lower than consolidation strain, because strain from soil is locally converted to shear deformation of the protective packaging [28]. There are two mechanisms of strain transfer between soil and polymer interfaces and they are influenced by stiffness of the polymer packaging (Young’s modulus and Poisson’s ratio) [29]. Soil particles tend to slide and scratch relatively hard polymer surface, while relatively soft surfaces deform and allow soil particles to roll [30]. Strain transfer is larger when particles slide. In our case, packaging is a soft polymer (teflon with a layer of silicone) and soil particles tend to roll, thus low strain transfer coefficient values are expected.

Friction coefficient between soil and packaging increases with normal pressure applied on the interface. In field conditions, this means that strain transfer coefficient increases with soil density [31]. Occasionally, stones and gravel of up to 10 cm size were discovered during the installation process and they were placed back into the borehole. Gravel pieces near the FBG sensors lowered the transfer of consolidation strain. FBG strain variation with depth is also caused by differences in the relative position of the fiber inside the tube. The shape of the teflon tube determines how much the fiber can adjust and release the strain.

### 3.2. Operation with Extraction from Well w1

**Figure 4:**
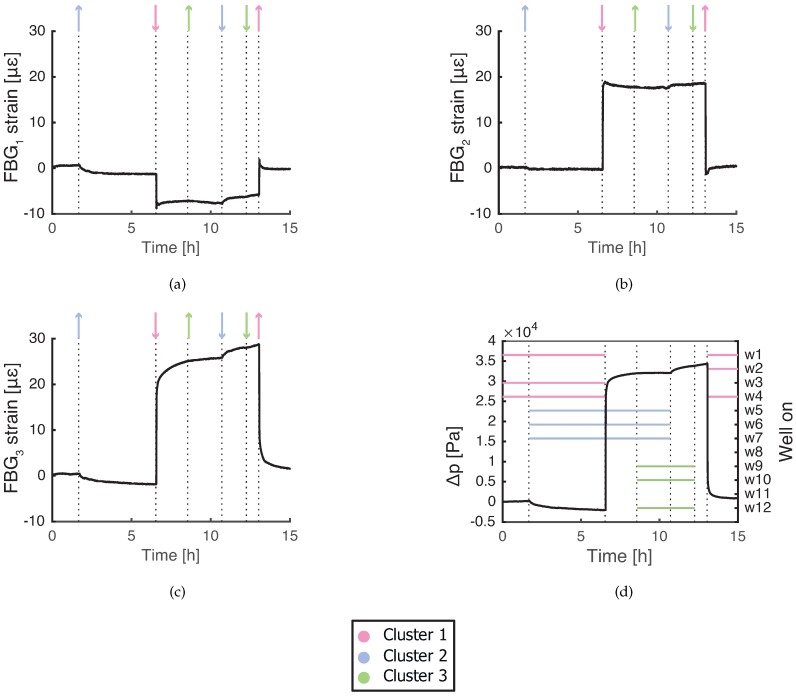
Measured strain response of FBG sensors during regular well operation with $w_{1}$ switched off and on: (**a**) $FBG_{1}$ at depth of 17.7 m; (**b**) $FBG_{2}$ at depth of 18.4 m; and (**c**) $FBG_{3}$ at depth of 19.1 m. (**d**) Pore pressure change in the reference piezometer in the 17.8–19.8 m depth range. ↑, a cluster of a corresponding color starts extracting; ↓, a cluster of a corresponding color stops extracting.

In the case $w_{1}$ is also extracting, the FBG sensors respond immediately to pore pressure changes, with amplitude decreasing with distance. Unlike in the previous case with $w_{1}$ off, the responses to Cluster 1 vary both in amplitude and shape (see Figure 4). The FBG responses to Cluster 2 follow the pore pressure changes in Figure 4d, while the response to Cluster 3 is barely visible for $FBG_{1}$ and $FBG_{3}$.

The extraction of $w_{1}$ clearly influences the FBG sensors. The amplitude of the fast response is approximately equal for start and stop of the Cluster 1 extraction. All soil strain is recovered after compression, which means that the consolidation is in the (primary) elastic stage. Sensors $FBG_{2}$ and $FBG_{3}$ show the fast response with the same sign as the fast pore pressure change, but they differ in slow relaxation behavior. The initial fast response of $FBG_{1}$ has an opposite sign compared to the other sensors. $FBG_{1}$ expands when the wells from the Cluster 1 are switched on and compresses at shutdown of the wells. This means that the pore pressure locally increases when the extraction of w1 starts. The explanation could be related to an occurrence of a clay layer near $FBG_{1}$. The discontinuities at the sand–clay boundary might cause local accumulation of groundwater and increase in the pore pressure. Compared to piezometers, FBG sensors can provide high resolution consolidation data from different depths and capture local heterogeneity of soil.

The observed FBG responses are not caused by volumetric changes of the fiber due to pore pressure or temperature (Table 1), FBG sensors most likely detect soil consolidation. When the extraction of $w_{1}$ is involved, the strain from soil is not always transferred with a constant scaling factor β. Various types of FBG relaxation responses to Cluster 1 are not caused by soil consolidation.

FBG relaxation response against the fast response is visible only for $FBG_{1}$ and $FBG_{2}$ in Figure 4. This response might be linked to the rate of soil compression/expansion. According to the results, if an FBG fiber in a teflon tube compresses/expands faster, it is more likely to show this relaxation behavior. Similar effects were previously observed by Drusová et al. [13] in an aquifer simulator filled with uniform sand. When pore pressure/flow suddenly changed, FBG sensors with PVC and teflon coating both showed a relaxation response against the pressure changes. It is known that polymers such as teflon and PVC behave differently based on the applied strain rate [32]. When a shock impulse is applied to these polymers, the relaxation response looks similar to what is measured by FBG sensors. In the case of teflon, this type of relaxation becomes visible with strain rates several orders of magnitude higher [33] than measured in this study. The maximal applied consolidation strain rate expected in this study was 57 με/s (calculated from the results in Figure 4d). Therefore, the consolidation strain rate is not a factor which determines the FBG response.

The relaxation response of FBG sensors in Figure 4 can be interpreted as decoupling of soil-packaging interface. Teflon tube slips in the soil and FBG fiber slips in the tube back to its original position after compression/expansion. The limit when the soil-packaging interface starts decoupling is determined by the friction coefficient and the magnitude of consolidation strain [34].

The larger magnitude of consolidation strain can explain why the relaxation behavior only occurs when $w_{1}$ start/stops extracting. In the case of extraction without $w_{1}$, the consolidation strain is not sufficient to cause the slip, the soil-packaging interface is fully coupled and consolidation strain is elastically transferred to FBG sensors.

According to Zhang et al. [34], if normal interface pressure is lower, friction coefficient is lower and packaging is more likely to decouple from soil. In the case of extraction with $w_{1}$, the soil-packaging friction coefficient can be influenced by groundwater flow. Groundwater flow close to an extraction well also has a vertical flow component and there is a possibility that flow channels are created along the packaging surface. This effect will decrease the coupling of the soil-packaging interface.

The interface decoupling limit has been reached by FBG1 and $FBG_{2}$. It is difficult to quantify the interface friction coefficient of the packaging in field conditions because it is influenced by the fiber installation process. The dependence on groundwater flow and soil type of the soil-packaging friction coefficient will be a subject of further research.

Several packaging options were already tested for FBG consolidation sensors. Wu et al. [29] used FBG fibers with polyurethane, metal sheath and steel conduit. Polyurethane seems to transfer the largest portion of consolidation strain, but it is not durable enough to be deployed at more than 100 m depth. Metal packaging can provide long-term protection of the fiber, but it will reduce the strain transmission [35]. Huang et al. [16] placed an FBG extensometer in a PVC tube and increased the friction with soil by adding friction rings. A teflon tube is a very suitable choice when it comes to protection of fibers in the subsurface because of its durability, low chemical reactivity and affordable price. A teflon tube is however not a suitable packaging for FBG consolidation sensors due to high strain losses. On the other hand, due to low friction coefficient, slow slip relaxation could become a new method to detect groundwater flow changes. An ideal packaging for this type of FBG groundwater flow and consolidation sensor would still be a teflon tube with a smaller diameter filled with elastic polymer to facilitate the strain transfer to the FBG sensors. The packaging should have a Young’s modulus lower or comparable to soil.

## 4. Conclusions

FBG sensors were deployed in an aquifer to find new methods to measure groundwater flow. The sensors measured soil consolidation caused by groundwater extraction from multiple wells. The magnitude of the FBG response to consolidation depends on the distance to extraction wells. Extraction of the nearest well causes the largest strain response, but nearby wells also need to be taken into account as they all influence the pore pressure in the aquifer. FBG sensors respond to an influence of wells from an area within 250 m radius (including Cluster 1, Cluster 2 and w9). The magnitude of FBG response at the start and stop of extraction are roughly equal, which means the soil deformation is elastic.

FBG packaging is an important factor which determines the FBG strain response. With our packaging (teflon tube), we measured consolidation strain one order of magnitude lower than the expected value. The influence of packaging on the FBG response is different based on magnitude of the pore pressure changes (groundwater flow). We compared two cases—with and without extraction of the nearest well.

When the nearest well was not extracting, the FBG sensors measured only soil consolidation caused by extraction from the further wells (starting from 80 m distance). The packaging in this case influenced the strain transfer magnitude between soil and FBG fiber. When the nearest well at 4 m distance started/stopped extracting, FBG fibers also showed a relaxation response influenced by a slow slip of the packaging in soil. The results suggest that friction coefficient between FBG packaging and soil is influenced by the groundwater flow, which was higher and not only in horizontal direction when the nearest well was extracting. FBG sensors can be used to measure soil consolidation with high resolution and show the occurrence of soil layers with different permeability.

A new packaging needs to be designed to increase the sensitivity of FBG sensors to consolidation strain and groundwater flow and to discriminate between them. More research needs to be conducted to translate the FBG data into groundwater flow, perhaps in combination with hydrological modeling tools. Once deployed in a well field, FBG sensors could measure continuously during regular operation of the wells. Long-term monitoring of consolidation and flow with high spatial resolution can show the first signs of clogging and identify the layers where it occurs. 

## Figures and Tables

**Table 1:** Maximal estimated FBG responses calculated from in-situ pore pressure and temperature data.

Without Extraction from $w_{1}$		With Extraction from $w_{1}$	
Measured Data		Measured Data	
max Δp	$6.6\times 10^{3}$ Pa	max Δp	$3.6\times 10^{4}$ Pa
max ΔT	0.5 K	max ΔT	0.1 K
Estimated FBG response		Estimated FBG response	
max $\Delta \lambda_{T}/\lambda_{0}$	3.7 × 10^−6^	max $\Delta \lambda_{T}/\lambda_{0}$	$0.7\times 10^{-6}$
max $\Delta \lambda_{c}/\lambda_{0}$	76.5 × 10^−6^ β	max $\Delta \lambda_{c}/\lambda_{0}$	$417.2\times 10^{-6}$ β
max $\Delta \lambda_{p}/\lambda_{0}$	−0.05 × 10^−6^ γ	max $\Delta \lambda_{p}/\lambda_{0}$	$-0.25\times 10^{-6}$ γ
Detection limit		Detection limit	
min $\Delta \lambda /\lambda_{0}$	0.65 × 10^−6^	min $\Delta \lambda /\lambda_{0}$	0.65 × 10^−6^

**Table 2:** Consolidation strain transfer coefficient for FBG sensors calculated for regular well operation without $w_{1}$ extracting.

	${\mathrm{FBG}}_{1}$	${\mathrm{FBG}}_{2}$	${\mathrm{FBG}}_{3}$
β	5.4 × 10^−2^	3.7 × 10^−2^	7.1 × 10^−2^

## Abbreviations

The following abbreviations are used in this manuscript:

FBG	Fiber Bragg grating
DTS	Distributed temperature sensing
PVC	Polyvinyl chloride

## References

[1] Revil A., Karaoulis M., Johnson T., Kemna A. (2012). Review: Some low-frequency electrical methods for subsurface characterization and monitoring in hydrogeology. Hydrogeol. J..

[2] Robert T.J.S. (2012). Geophysical Identification, Characterization, and Monitoring of Preferential Groundwater Flow Paths in Fractured Media. Ph.D. Thesis.

[3] Bayless E.R., Wayne A., Mandell J.R.U. (2011). Accuracy of Flowmeters Measuring Horizontal Groundwater Flow in an Unconsolidated Aquifer Simulator. Ground Water Monit. Remediat..

[4] Bense V.F., Read T., Bour O., Le Borgne T., Coleman T., Krause S., Chalari A., Mondanos M., Ciocca F., Selker J.S. (2016). Distributed Temperature Sensing as a downhole tool in hydrogeology. Water Resour. Res..

[5] Anderson M.P. (2005). Heat as a ground water tracer. Ground Water.

[6] Read T., Bour O., Bense V., Borgne T.L., Goderniaux P., Klepikova M.V., Hochreutener R., Lavenant N., Boschero V. (2013). Characterizing groundwater flow and heat transport in fractured rock using fiber-optic distributed temperature sensing. Geophys. Res. Lett..

[7] Klepikova M.V., Borgne T.L., Bour O., Dentz M. (2016). Heat as a tracer for understanding transport processes in fracturedmedia: Theory and field assessment from multiscale thermal push-pull tracer tests. Water Resour. Res..

[8] Read T., Bense V.F., Hochreutener R., Bour O., Le Borgne T., Lavenant N., Selker J.S. (2015). Thermal-plume fibre optic tracking (T-POT) test for flow velocity measurement in groundwater boreholes. Geosci. Instrum. Methods Data Syst..

[9] Briggs M.A., Buckley S.F., Bagtzoglou A.C., Werkema D.D., Lane J.W. (2016). Actively heated high-resolution fiber-optic-distributed temperature sensing to quantify streambed flow dynamics in zones of strong groundwater upwelling. Water Resour. Res..

[10] Somogyvári M., Bayer P., Brauchler R. (2016). Travel-time-based thermal tracer tomography. Hydrol. Earth Syst. Sci..

[11] Bakx W., Doornenbal P.J., van Weesep R.J., Bense V.F., Oude Essink G.H.P., Bierkens M.F.P. (2019). Determining the Relation between Groundwater Flow Velocities and Measured Temperature Differences Using Active Heating-Distributed Temperature Sensing. Water.

[12] Des Tombe B.F., Bakker M., Smits F., Schaars F., van der Made K.J. (2019). Estimation of the Variation in Specific Discharge Over Large Depth Using Distributed Temperature Sensing (DTS) Measurements of the Heat Pulse Response. Water Resour. Res..

[13] Drusová S., Bakx W., Wexler A.D., Offerhaus H.L. (2019). Possibilities for Groundwater Flow Sensing with Fiber Bragg Grating Sensors. Sensors.

[14] Gambolati G., Teatini P. (2015). Geomechanics of subsurface water withdrawal and injection. Water Resour. Res..

[15] Zhu H.H., Shi B., Zhang C.C. (2017). FBG-Based Monitoring of Geohazards: Current Status and Trends. Sensors.

[16] Huang A.B., Wang C.C., Lee J.T., Ho Y.T. (2016). Applications of FBG-based sensors to ground stability monitoring. J. Rock Mech. Geotech. Eng..

[17] Schenato L. (2017). A Review of Distributed Fibre Optic Sensors for Geo-Hydrological Applications. Appl. Sci..

[18] Wang J., Jiang L., Sun Z., Hu B., Zhang F., Song G., Liu T., Qi J., Zhang L. (2017). Research on the Surface Subsidence Monitoring Technology Based on Fiber Bragg Grating Sensing. Photonic Sens..

[19] Loáiciga H.A. (2012). Consolidation Settlement in Aquifers Caused by Pumping. J. Geotech. Geoenviron. Eng..

[20] Niu W., Wang Z., Chen F., Li H. (2013). Settlement Analysis of a Confined Sand Aquifer Overlain by a Clay Layer due to Single Well Pumping. Math. Probl. Eng..

[21] Biot M.A. (1955). Theory of elasticity and consolidation for a porous anisotropic solid. J. Appl. Phys..

[22] Shen S.L., Xu Y.S. (2011). Numerical evaluation of land subsidence induced by groundwater pumping in Shanghai. Can. Geotech. J..

[23] Shen S.L., Ma L., Xu Y.S., Yin Z.Y. (2013). Interpretation of increased deformation rate in aquifer IV due to groundwater pumping in Shanghai. Can. Geotech. J..

[24] Look B. (2007). Handbook of Geotechnical Investigation and Design Tables.

[25] Sheng H.J., Fu M.Y., Chen T.C., Liu W.F., Bor S.S. (2004). A Lateral Pressure Sensor Using a Fiber Bragg Grating. IEEE Photonics Technol. Lett..

[26] Li D., Ren L., Li H., Yasin D.M. (2012). Mechanical Property and Strain Transferring Mechanism in Optical Fiber Sensors. Fiber Optic Sensors.

[27] Cadusch P.J., Thompson A.C., Stoddart P.R., Wade S.A. (2012). Modeling of Bend Effects on Fiber Bragg Gratings Modeling of Bend Effects on Fiber Bragg Gratings. Proc. SPIE Int. Soc. Opt. Eng..

[28] Her S.C., Huang C.Y. (2011). Effect of Coating on the Strain Transfer of Optical Fiber Sensors. Sensors.

[29] Wu J., Jiang H., Su J., Shi B., Jiang Y., Gu K. (2015). Application of distributed fiber optic sensing technique in land subsidence monitoring. J. Civ. Struct. Health Monit..

[30] O’Rourke T.D., Druschel S.J., Netravali A.N. (1990). Shear strength characteristics of sand-polymer interfaces. J. Geotech. Eng..

[31] Fuggle A., Frost J. Particle size effects in interface shear behavior and geomembrane wear. Proceedings of the International Symposium on Characterization and Behavior of Interfaces.

[32] Bourne N.K. (2016). On the Shock Response of Polymers to Extreme Loading. J. Dyn. Behav. Mater..

[33] Walley S.M., Field J.E. (1994). Strain rate sensitivity of polymers in compression from low to high rates. DYMAT J..

[34] Zhang C.C., Zhu H.H., She J.K., Zhang D., Shi B. (2015). Quantitative evaluation of optical fiber/soil interfacial behavior and its implications for sensing fiber selection. IEEE Sens. J..

[35] Lin Y.B., Chang K.C., Chern J.C., Wang L.A. (2016). Packaging Methods of Fiber-Bragg Grating Sensors in Civil Structure Applications. IEEE Sens. J..

